# Supplementary material for: The Systems Biology Research Tool: evolvable open-source software
Source: BMC Syst Biol. 2008 Jun 29;2:55. doi: 10.1186/1752-0509-2-55 (PMC2446383; doi:10.1186/1752-0509-2-55)
Supplement: Additional file 1 — SBRT Archive. An archive of the current version of the Systems Biology Research Tool. [file 1752-0509-2-55-S1.zip › sbrt-1.4.0/doc/developers_guide/api/sbrt/kernel/fba/comp/package-tree.html]

sbrt.kernel.fba.comp Class Hierarchy


|  |  |  |
| --- | --- | --- |
| **Overview** \| **Package** \| Class \| Use \| **Tree** \| **Deprecated** \| **Index** \| **Help** | |  |
| **PREV**   **NEXT** | **FRAMES**    **NO FRAMES**     **All Classes** |  |


---


## Hierarchy For Package sbrt.kernel.fba.comp

**Package Hierarchies:**: All Packages

---

## Class Hierarchy

- java.lang.Object
  - sbrt.kernel.fba.comp.**AbstractCvComputer** (implements sbrt.kernel.fba.comp.FluxCvComputer)
    - sbrt.kernel.fba.comp.**AbstractFluxIntervalComputer** (implements sbrt.kernel.fba.comp.FluxIntervalComputer)
      - sbrt.kernel.fba.comp.**FastMsFluxRangeComputer**- sbrt.kernel.fba.comp.**MsFluxIntervalComputer**- sbrt.kernel.fba.comp.**ReverseRxnFluxIntervalComputer**- sbrt.kernel.fba.comp.**SimpleFluxIntervalComputer**
      - sbrt.kernel.fba.comp.**CdHarFluxVectorComputer**- sbrt.kernel.fba.comp.**RxnDeletionComputer**
    - sbrt.kernel.fba.comp.**CdHarFluxVectorComputer.Results**- sbrt.kernel.fba.comp.**FbaOptComputer** (implements sbrt.kernel.fba.comp.FluxCvComputer)- sbrt.kernel.fba.comp.**FbaOptComputer.Results**- sbrt.kernel.fba.comp.**FbaSolver**- sbrt.kernel.fba.comp.**FluxCapComputer**- sbrt.kernel.fba.comp.**InitialPointComputer** (implements sbrt.kernel.fba.comp.FluxCvComputer)- sbrt.kernel.fba.comp.**InitialPointComputer.Results**- sbrt.kernel.fba.comp.**MsFluxomeReducer** (implements sbrt.kernel.fba.comp.FluxomeReducer<T>)- sbrt.kernel.fba.comp.**SlpCycleComputer** (implements sbrt.kernel.fba.comp.CycleComputer)- sbrt.kernel.fba.comp.**SlpExCurrComputerV2** (implements sbrt.kernel.fba.comp.ExtremeCurrentComputer)- java.lang.Throwable (implements java.io.Serializable)
                          - java.lang.Exception
                            - java.lang.RuntimeException
                              - sbrt.kernel.fba.comp.**FbaOptException**- sbrt.kernel.fba.comp.**FluxCapException**- sbrt.kernel.fba.comp.**FluxRangeException**- sbrt.kernel.fba.comp.**InvalidFluxVectorException**
                          - sbrt.kernel.fba.comp.**WwFluxomeReducer** (implements sbrt.kernel.fba.comp.FluxomeReducer<T>)

## Interface Hierarchy

- sbrt.kernel.fba.comp.**CycleComputer**- sbrt.kernel.fba.comp.**ExtremeCurrentComputer**- sbrt.kernel.fba.comp.**FluxCvComputer**
      - sbrt.kernel.fba.comp.**FluxIntervalComputer**
      - sbrt.kernel.fba.comp.**FluxomeReducer**<T>

---


|  |  |  |
| --- | --- | --- |
| **Overview** \| **Package** \| Class \| Use \| **Tree** \| **Deprecated** \| **Index** \| **Help** | |  |
| **PREV**   **NEXT** | **FRAMES**    **NO FRAMES**     **All Classes** |  |


---
